# *Sorobiellomyces jilinensis* gen. et sp. nov. (*Polycephalomycetaceae*) and *Niveomyces pseudoalbus* sp. nov. (*Cordycipitaceae*): Two hyperparasitic fungi associated with *Ophiocordyceps*

**DOI:** 10.3897/mycokeys.134.194187

**Published:** 2026-06-12

**Authors:** Xian Zhang, Xing-Can Peng, Ausana Mapook, Kevin D. Hyde, De-Ping Wei, Zheng-Hui Liu, Natsaran Saichana, Ting-Chi Wen

**Affiliations:** 1 School of Science, Mae Fah Luang University, Chiang Rai 57100, Thailand Center of Excellence in Fungal Research, Mae Fah Luang University Chiang Rai Thailand; 2 State Key Laboratory of Green Pesticide, Key Laboratory of Green Pesticide and Agricultural Bioengineering, Ministry of Education, Guizhou University, Guiyang 550025, China School of Science, Mae Fah Luang University Chiang Rai Thailand; 3 Engineering Research Center of Southwest Bio-Pharmaceutical Resources, Ministry of Education, Guizhou University, Guiyang 550025, China Engineering Research Center of Southwest Bio-Pharmaceutical Resources, Ministry of Education, Guizhou University Guiyang China; 4 Center of Excellence in Fungal Research, Mae Fah Luang University, Chiang Rai 57100, Thailand Key Laboratory of Green Pesticide and Agricultural Bioengineering, Ministry of Education, Guizhou University Guiyang China

**Keywords:** *
Cordycipitaceae
*, entomopathogenic fungi, hyperparasitism, morphology, phylogenetics, *
Polycephalomycetaceae
*

## Abstract

Hyperparasitism in fungi remains poorly understood and documented, despite its ecological significance. In this study, a hyperparasitic fungus associated with *Ophiocordyceps jilinensis* was collected in Jilin Province, China. A new genus, *Sorobiellomyces* gen. nov., is proposed to accommodate *Sorobiellomyces jilinensis* sp. nov., which is parasitic on an entomopathogenic host. Morphological observations and multilocus phylogenetic analyses based on ITS, SSU, LSU, *tef*1-α, *rpb*1, and *rpb*2 sequence data placed *S. jilinensis* within *Polycephalomycetaceae* as a distinct lineage. *Sorobiellomyces* is characterized by a white to yellowish hyphal covering, absence of synnemata, and cylindrical to subulate phialides producing hyaline, ovoid conidia. *Ophiocordyceps jilinensis* is redescribed and illustrated. In addition, *Niveomyces pseudoalbus* sp. nov. is introduced based on morphological and phylogenetic evidence. These results expand the diversity of *Polycephalomycetaceae* and *Cordycipitaceae* and provide new data on fungal hyperparasitism.

## Introduction

Hyperparasitism, a phenomenon in which a parasite is itself parasitized by another organism, represents a complex multitrophic interaction that plays a significant yet understudied role in ecological and evolutionary dynamics (Lebert 2020). The term was first introduced by Boosalis (1964) to describe fungi parasitic on other fungi, particularly when the host is also a parasite. This distinguishes hyperparasites from broader mycoparasites, which may target saprotrophic, mutualistic, or pathogenic fungi without restriction to host trophic status (Karlsson et al. 2017; Moore et al. 2020). Hyperparasitic interactions involve at least three trophic levels: a primary host, a primary parasite, and a secondary parasite, the hyperparasite (Bermúdez-Cova et al. 2022). These interactions often negatively affect host fitness, thereby influencing population regulation and disease transmission in natural and agricultural systems (Bermúdez-Cova et al. 2022).

Hyperparasitism is widespread across diverse organisms, including insect parasitoids, viruses, and parasitic plants (Sullivan 1987; Grybchuk et al. 2018; Sun et al. 2019; Krasylenko et al. 2021). Among fungi, hyperparasites are taxonomically diverse, occurring in *Cryptomycota*, early-diverging lineages of *Zygomycetes*, *Basidiomycota*, and various groups of *Ascomycota* (Jeffries 1985; Lutz et al. 2004; Gleason et al. 2012). They often exhibit high host specificity, targeting particular structures such as sclerotia, spores, or sporocarps of their fungal hosts (Kepler et al. 2012a, 2013; Berndt 2013). Notable examples include species of *Trichoderma (Hypocreales)*, which are well-known mycoparasites of plant pathogens, as well as numerous hypocrealean fungi parasitizing parasites of plants, animals, or other fungi (Elad et al. 1980; Sung et al. 2007; Brotman et al. 2010; Wijayawardene et al. 2022). Members of *Dothideomycetes* also include prominent hyperparasites, such as *Ampelomyces* on powdery mildews and *Cladosporium* on diverse parasitic hosts (Kiss et al. 2004; Moricca et al. 2005). Hyperparasitism has also been reported in hypocrealean entomopathogenic systems, such as species of *Niveomyces*, which have been documented as hyperparasites of Ophiocordyceps camponoti-floridani (Araújo et al. 2018, 2020, 2022; Lebert 2020).

Various hypocrealean entomopathogenic fungi are known to parasitize species of *Ophiocordyceps*, particularly those within *Polycephalomycetaceae*. Xiao et al. (2023) established the family *Polycephalomycetaceae*, segregating it from *Ophiocordycipitaceae* based on multilocus phylogenetic analyses, to accommodate the genera *Polycephalomyces*, *Perennicordyceps*, and *Pleurocordyceps* (Hyde et al. 2024). Subsequently, Johnston and Park (2023) introduced the genus *Dingleyomyces*, assigning *Dingleyomyces lloydii* to *Polycephalomycetaceae* based on combined morphological and phylogenetic evidence. Wang et al. (2024) further proposed the new genus *Paradingleyomyces* to accommodate *Paradingleyomyces lepidopterorum*, which forms a distinct monophyletic lineage in multigene phylogenetic analyses and is morphologically characterized by a unique stromal subiculum and perithecial arrangement. Members of *Polycephalomycetaceae* are frequently hyperparasitic. For instance, Liu et al. (2024a) reported four species of *Pleurocordyceps* as hyperparasitic on *Ophiocordyceps*, while *Pleurocordyceps parvicapitata* infects *Perennicordyceps elaphomyceticola*. Xiao et al. (2023) noted that two species of *Perennicordyceps* parasitize *Ophiocordyceps*, with *Perennicordyceps cuboidea* even occurring on a *Cordyceps* species. The monotypic *Dingleyomyces lloydii* also parasitizes *Ophiocordyceps* sp. (Johnston and Park 2023), and species of *Torrubiellomyces* have been identified as obligate parasites of Ophiocordyceps camponoti-floridani (Araújo et al. 2022).

*Niveomyces* is a genus of hyperparasitic fungi associated with hypocrealean entomopathogens, particularly the well-known “zombie-ant” fungi in *Ophiocordyceps*. Following the establishment of *Niveomyces* (type species *Niveomyces coronatus*) by Araújo et al. (2022), Kobmoo et al. (2023) expanded the genus by describing five additional species: *Niveomyces albus*, *N. formicidarum*, *N. hirsutellae*, *N. insectorum*, and *N. multisynnematus*. Ecologically, *Niveomyces coronatus* parasitizes Ophiocordyceps camponoti-floridani, a pathogen that manipulates the carpenter ant *Camponotus floridanus*, as well as related lineages infecting ants across the Americas. However, the host range of *Niveomyces* is broader than previously recognized. Species described from Thailand parasitize diverse hypocrealean entomopathogens across multiple insect orders, including *Ophiocordyceps dipterigena* on *Diptera*, *Ophiocordyceps polyrhachis-furcata* on *Polyrhachis furcata* (*Formicidae*), *Hirsutella aff. versicolor* on *Hemiptera (Cicadellidae)*, and *Ophiocordyceps aff. flavida* on additional leafhopper hosts (Kobmoo et al. 2023). In Africa, *Niveomyces insectorum* has been recorded from *Paltothyreus tarsatus* associated with *Gibellula formicarum* (De Hoog 1974). These findings indicate that *Niveomyces* species are widespread, infect a taxonomically diverse range of entomopathogenic fungi, and occur across varied ecological settings. This suggests that hyperparasitism within this lineage occurs in varied ecological settings.

In this study, two novel hyperparasitic fungal specimens were collected from different regions of China. The first specimen, collected from Liaoning Province, was identified as a member of the family *Polycephalomycetaceae* and was found parasitic on *Ophiocordyceps jilinensis*, which was collected concurrently. The second specimen, collected from Yunnan Province, also parasitizes a species of *Ophiocordyceps*. To determine their taxonomic placement, morphological examinations and multilocus phylogenetic analyses were conducted based on ITS, SSU, LSU, *tef*1-α, *rpb*1, and *rpb*2 sequence data. These results provide new data on fungal–fungal interactions, particularly involving hyperparasitic and entomopathogenic fungi.

## Materials and methods

### Collection and morphological study

Entomopathogenic fungal samples were collected from humid, low-sunlight evergreen and deciduous forest habitats in Liaoning and Yunnan provinces, China. Fungal specimens were primarily obtained from the upper and lower surfaces of living leaves. First, fresh specimens were photographed with a mobile phone (Huawei P40) to document their natural states. Then, the samples were carefully placed in plastic containers to preserve their structural integrity during transport to the laboratory. Macro- and microscopic structures were observed with a stereomicroscope (Leica S9E or Olympus SZ61) and photographed using a compound microscope (Leica DM2500 or Nikon ECLIPSE Ni). Key structures, including synnemata, conidiophores, phialides, and conidia, were photographed and measured using integrated digital imaging systems and software (Leica imaging software, Tarosoft Image Framework, or Adobe Photoshop). Specimens were preserved dry with silica gel and deposited in the Herbarium of Cryptogams at the Kunming Institute of Botany, Chinese Academy of Sciences (HKAS). Specimens and sequences were registered in Index Fungorum (IF) following standard procedures.

### DNA extraction, PCR amplification, and sequencing

Genomic DNA was extracted from dried fungal specimens using the Omega Fungus Genomic DNA Extraction Kit (China) according to the manufacturer’s instructions. PCR amplification was performed using six genetic regions (ITS, LSU, SSU, *tef*1-α, *rpb*1, and *rpb*2) and following the methodology of Wang et al. (2024). The corresponding primers and amplification conditions followed those detailed by Peng et al. (2024). The PCR products were sequenced by Tsingke Biological Technology (Chongqing, China). All newly generated sequences were deposited in GenBank and used for phylogenetic reconstruction. Relevant sequence information, including accession numbers, is compiled in Tables 1, 2.

**Table 1.** GenBank accession numbers of the taxa within *Ophiocordycipitaceae* and *Polycephalomycetaceae* used in the phylogenetic analyses. The newly generated sequences are in bold; ^T^ represents type strains, type specimens, or neotypes.

Taxa	Voucher	ITS	SSU/18s	LSU/28s	*tef*1-*α*	*rpb*1	*rpb*2	References
*Cordyceps pleuricapitata*	NBRC 100745	JN943304	JN941750	JN941391	KF049679	JN992484	KF049667	Kepler et al. (2013)
*C. pleuricapitata*	NBRC 100746	JN943306	JN941749	JN941392	KF049680	JN992483	KF049668	Kepler et al. (2013)
*Dingleyomyces lloydii*	PDD 121254	OR602634	OR647563	OR602640	OR588853	OR588860	OR588858	Johnston and Park (2023)
*Hirsutella illustris*	ARSEF 5539	KM652160	KM652069	AY518380	KM651996	KM652037		Simmons et al. (2015)
*H. kirchneri*	ARSEF 5551	KM652161	KM652070	KM652113	KM651997			Simmons et al. (2015)
*H. lecaniicola*	ARSEF 8888	KM652162	KM652071	KM652114	KM651998	KM652038		Simmons et al. (2015)
*H. sinensis*	HMAS 55469	AJ243980						Zhao et al. (1999)
*H. versicolor*	ARSEF 1037		KM652102	KM652150	KM652029	KM652063		Simmons et al. (2015)
*Ophiocordyceps appendiculata*	NBRC 106960	JN943326	JN941728	JN941413	AB968577	JN992462	AB968539	Ban et al. (2015)
*O. arborescens*	NBRC 105891	AB968398	AB968386	AB968414	AB968572		AB968534	Ban et al. (2015)
*O. brunneinigra*	BCC 69015			MF614653	MF614637		MF614680	Luangsa-Ard et al. (2018)
*O. citrina*	TNS F18537			KJ878903	KJ878983			Quandt et al. (2014)
*O. coccidiicola*	NBRC 100682	AB968404	AB968391	AB968419	AB968583		AB968545	Ban et al. (2015)
*O. crinalis*	GDGM 17327		KF226253	KF226254	KF226256	KF226255		Wang et al. (2014)
*O. issidarum*	MFLU 17-0751^T^	MF398185		MF398188				Hyde et al. (2017)
*O. jilinensis*	HKAS 144583^T^	PQ528946	PQ528940	PQ528943	PQ522478	PQ522476	PQ569911	Cao et al. (2025)
*O. jilinensis*	HKAS 144584	PQ528947	PQ528941	PQ528944	PQ522479		PQ569912	Cao et al. (2025)
*O. jilinensis*	**HKAS 149968**	**PX900240**	**PX906583**			**PX896350**		**This study**
*O. macroacicularis*	NBRC 100685			AB968416	AB968574		AB968536	Ban et al. (2015)
*O. nujiangensis*	YFCC 8880^T^		ON723384	ON723381	ON868820	ON868823	ON868826	Sun et al. (2022)
*O. rubiginosiperitheciata*	NBRC 100946	JN943341	JN941705	JN941436	AB968581	JN992439	AB968543	Ban et al. (2015)
*O. sinensis*	EFCC 7287	JN049854	EF468971	EF468827	EF468767	EF468874	EF468924	Sung et al. (2007)
*O. spataforae*	NHJ 12525		EF469125	EF469078	EF469063	EF469092	EF469111	Sung et al. (2007)
*O. stylophora*	OSC 111000	JN049828	DQ522552	DQ518766	DQ522337	DQ522382	DQ522433	Spatafora et al. (2007)
*O. xuefengensis*	GZUHHN13	KC631804	KC631785		KC631790	KC631795		Wen et al. (2013)
*Paradingleyomyces lepidopterorum*	HKAS 131926^T^	OR878363		OR828238		OR829674	OR880683	Wang et al. (2024)
*Pa. lepidopterorum*	HKAS 131927	OR878364		OR828239	OR880679	OR829675		Wang et al. (2024)
*Perennicordyceps cuboidea*	NBRC 100941	JN943329	JN941725	JN941416		JN992459		Schoch et al. (2012)
*Pe. elaphomyceticola*	NTUCC 17-021^T^	MK840823		MK840812	MK839229	MK839220	MK839211	Xiao et al. (2023)
*Pe. lutea*	KUMCC 3004			OQ474910				Xiao et al. (2023)
*Pe. paracuboidea*	NBRC 101742	JN943338	JN941710	JN941431	KF049685	JN992444	KF049669	Schoch et al. (2012)
*Pe. prolifica*	NBRC 103838	JN943339	JN941707	JN941434		JN992441		Schoch et al. (2012)
*Pe. ryogamiensis*	NBRC 101751	JN943343	JN941703	JN941438	KF049688	JN992437		Schoch et al. (2012)
*Pe. zongqii*	DY05421^T^	PQ211278		PQ211282	PQ223679		PQ223677	Chen et al. (2024)
*Pleurocordyceps agarica*	YHHPA1305	KP276651	KP276655		KP276659	KP276663	KP276667	Wang et al. (2015b)
*Pl. aurantiaca*	MFLUCC 17-2113^T^	MG136916	MG136904	MG136910	MG136875	MG136866	MG136870	Xiao et al. (2019)
*Pl. clavisynnema*	GZLG 23-102^T^	OQ968788		OQ968796	OQ982009			Xiao et al. (2024)
*Pl. fusiformispora*	YFCC 07239279^T^	PP002030		PP410610	PP254877	PP581807	PP581824	Liu et al. (2024a)
*Pl. heilongtanensis*	KUMCC 3008	OQ172091	OQ172111	OQ172063	OQ459731	OQ459759	OQ459805	Xiao et al. (2023)
*Pl. lanceolata*	GACP 17-2004^T^	OQ172076	OQ172110	OQ172046	OQ459726	OQ459754	OQ459800	Xiao et al. (2023)
*Pl. lianzhouensis*	GIMYY9603	EU149922	KF226249	KF226250	KF226252	KF226251		Xiao et al. (2023)
*Pl. litangensis*	YFCC 06109293^T^	PP410597	PP541902	PP410593	PP550103	PP697751		Liu et al. (2024b)
*Pl. marginaliradians*	MFLU 17-1582^T^	MG136920	MG136908	MG136914	MG136878	MG136869	MG271931	Xiao et al. (2019)
*Pl. neoagarica*	GZLG 23-103^T^	OQ968790		OQ968795				Xiao et al. (2024)
*Pl. nipponicus*	BCC_2325	KF049665	KF049622	KF049640	KF049696	KF049655	KF049677	Kepler et al. (2013)
*Pl. nutansis*	MFLU 21-0275^T^	OQ172073	OQ172119	OQ172048	OQ459739	OQ459765	OQ459811	Xiao et al. (2023)
*Pl. onorei*	BRA CR23902^T^	KU898841						Crous et al. (2017)
*Pl. parvicapitata*	MFLU 21-0270^T^	OQ172082	OQ172105	OQ172054	OQ459722	OQ459751	OQ459796	Xiao et al. (2023)
*Pl. phaothaiensis*	BCC84551	MF959731		MF959735	MF959739	MF959743		Crous et al. (2017)
*Pl. ramosopulvinata*	SU 65			DQ118742	DQ118753	DQ127244		Chaverri et al. (2005)
*Pl. sanduensis*	GZLG 23-104^T^	OQ968786		OQ968798	OQ982005		OQ982000	Xiao et al. (2024)
*Pl. sinensis*	CN 80-2	HQ832884	HQ832887	HQ832886	HQ832890	HQ832888	HQ832889	Wang et al. (2012)
*Pl. vitellina*	KUMCC 3006^T^	OQ172089		OQ172061	OQ459729	OQ459757	OQ459803	Xiao et al. (2023)
*Pl. yunnanensis*	YHCPY 1005	KF977848			KF977850	KF977852	KF977854	Wang et al. (2015a)
*Polycephalomyces albiramus*	GACP 21-XS08^T^	OQ172092	OQ172115	OQ172037	OQ459735	OQ459761	OQ459807	Xiao et al. (2023)
*Po. albiramus*	GACPCC 21-XS08^T^	OQ172093	OQ172116	OQ172038	OQ459734	OQ459762	OQ459808	Xiao et al. (2023)
*Po. formosus*	NBRC 100686	MN586830	MN586821	MN586839	MN598054	MN598045	MN598061	Wang et al. (2021)
*Po. formosus*	CGMCC 5.2207	MN586834	MN586825	MN586843	MN598058	MN598049	MN598065	wang et al. (2021)
*Po. jinghongensis*	YFCC 02959283^T^	PP274089	PP274093	PP274109	PP581803	PP697747	PP581819	Liu et al. (2024a)
*Po. jinghongensis*	YFCC 02959284	PP274090	PP274094	PP274110	PP581804	PP697748	PP581820	Liu et al. (2024a)
*Po. multiperitheciatae*	YFCC 06149287^T^	PP274102	PP274108	PP274118	PP581802		PP581818	Liu et al. (2024a)
*Po. multiperitheciatae*	YFCC 06149288	PP274098	PP274104	PP274114	PP581798	PP697743	PP581815	Liu et al. (2024a)
*Po. myrmecophilus*	YFCC 09289443^T^	PP410602	PP410608	PP410605	PP581795	PP697740	PP581812	Liu et al. (2024a)
*Po. myrmecophilus*	YFCC 09289444	PP410603	PP410609	PP410606	PP581796	PP697741	PP581813	Liu et al. (2024a)
*Po. tengchongensis*	HKAS 131923^T^	OR878365	PP129612	OR828240		OR829676	OR880685	Wang et al. (2024)
***Sorobiellomyces jilinensis***	**HKAS 149967^T^**	**PX900238**	**PX906581**	**PX900234**	**PX896355**	**PX896348**	**PX896353**	**This study**
*S. jilinensis*	**HKAS 152704**	**PX900239**	**PX906582**	**PX900235**	**PX896356**	**PX896349**	**PX896354**	**This study**
*Torrubiellomyces zombiae*	NY04434801^T^		ON493543	ON493602	ON513396	ON513398	ON513402	Araújo et al. (2022)
*T. zombiae*	FieldB		ON493544	ON493603	ON513395			Araújo et al. (2022)
*T. zombiae*	Polyceph			ON493607	ON513394			Araújo et al. (2022)
*Cordyceps militaris*	OSC 93623	JN049825	AY184977	AY184966	DQ522332	DQ522377	AY545732	Kepler et al. (2012a)
*C. militaris*	YFCC 6587		MN576762	MN576818	MN576988	MN576878	MN576932	Wang et al. (2020)

**Table 2.** GenBank accession numbers of the taxa within *Cordycipitaceae* used in the phylogenetic analyses. The newly generated sequences are in bold; ^T^ represents type strains, type specimens, or neotypes.

Taxa	Voucher	ITS	SSU/18s	LSU/28s	*tef*1-*α*	*rpb*1	*rpb*2	References
*Akanthomyces aculeatus*	HUA 186145^T^		MF416572	MF416520	MF416465			Kepler et al. (2017)
*Ak. sulphureus*	TBRC 7248^T^			MF140722	MF140843	MF140787	MF140812	Mongkolsamrit et al. (2018)
*Ascopolyporus polychrous*				DQ118737	DQ118745	DQ127236		Chaverri et al. (2005)
*As. villosus*	ARSEF 6355			AY886544	DQ118750	DQ127241		Chaverri et al. (2005)
*Beauveria bassiana*	ARSEF 1564^T^	HQ880761			HQ880974	HQ880833	HQ880905	Rehner et al. (2011)
*Be. bassiana*	ARSEF 7518	HQ880762			HQ880975	HQ880834	HQ880906	Rehner et al. (2011)
*Bhushaniella rubra*	BCC47541^T^			OQ892133	OQ914428	OQ914431	OQ914433	Mongkolsamrit et al. (2023)
*Bh. rubra*	BCC47542			OQ892134	OQ914429	OQ914432	OQ914434	Mongkolsamrit et al. (2023)
*Blackwellomyces cardinalis*	OSC 93609^T^		AY184973	AY184962	DQ522325	DQ522370	DQ522422	Spatafora et al. (2007)
*Bl. cardinalis*	OSC 93610	JN049843	AY184974	AY184963	EF469059	EF469088	EF469106	Sung et al. (2007), Kepler et al. (2012a)
*Cordyceps militaris*	OSC 93623	JN049825	AY184977	AY184966	DQ522332	DQ522377	AY545732	Sung et al. (2007), Kepler et al. (2012b)
*Cord. militaris*	YFCC 6587		MN576762	MN576818	MN576988	MN576878	MN576932	Wang et al. (2020)
*Corniculantispora aranearum*	CBS 797.84^T^			KM283787	KM283811	KM283833	KM283853	Evans et al. (2025)
*Corpulentispora magnispora*	CGMCC3.19304^T^			MK329007	MK336037		MK335985	Zhang et al. (2021)
*Corp. magnispora*	LC12469			MK329008	MK336038		MK335986	Zhang et al. (2021)
*Engyodontium parvisporum*	IHEM 22910^T^	LC092896		LC092915	LC425558			Tsang et al. (2016)
*E. rectidentatum*	CBS 641.74	LC092895		LC092914	LC425540			Tsang et al. (2016)
*Flavocillium bifurcatum*	YFCC 6101^T^	MN576833	MN576725	MN576781	MN576951	MN576841	MN576897	Wang et al. (2020)
*Gamszarea humicola*	CGMCC 3.19303^T^	MK329092	MK311230	MK328997	MK336027		MK335979	Zhang et al. (2021)
*Ga. wallacei*	CBS 101237	EF513022	AY184978	AY184967	EF469073	EF469102	EF469119	Sung et al. (2007)
*Gibellula gamsii*	BCC 27968^T^	MH152529		MH152539	MH152560	MH152547		Kuephadungphan et al. (2019)
*Gi. gamsii*	BCC 28797	MH152531		MH152541	MH152562	MH152549	MH152557	Kuephadungphan et al. (2019)
*Hevansia novoguineensis*	CBS 610.80^T^	MH532831		MH394646	MH521885		MH521844	Mongkolsamrit et al. (2020)
*H. novoguineensis*	BCC 42675	MZ684089		MZ684004	MZ707814		MZ707835	Mongkolsamrit et al. (2020)
*Jenniferia thomisidarum*	BCC 37881^T^	MZ684099		MZ684010	MZ707823	MZ707830	MZ707843	Mongkolsamrit et al. (2020)
*J. thomisidarum*	BCC 37882	MZ684100		MZ684011	MZ707824	MZ707831	MZ707844	Mongkolsamrit et al. (2020)
*Kanoksria zaquensis*	HMAS 246915^T^			MT789697	MT797812	MT797810		Khonsanit et al. 2024
*K. zaquensis*	HMAS 246917			MT789696	MT797811	MT797809		Khonsanit et al. 2024
*Lecanicillium antillanum*	CBS 350.85	NR_111097	AF339585	AF339536	DQ522350	DQ522396	DQ522450	Sung et al. (2001), Spatafora et al. (2007)
*Le. tenuipes*	CBS 309.85	JN036556	KM283778	KM283802	DQ522341	KM283844	KM283866	Zhou et al. (2022)
*Liangia sinensis*	YFCC 3103^T^	MN576831	MN576726	MN576782	MN576952	MN576842	MN576898	Wang et al. (2020)
*Li. sinensis*	YFCC 3104	MN576832	MN576727	MN576783	MN576953	MN576843	MN576899	Wang et al. (2020)
*Neohyperdermium piperis*	CBS 116719			AY466442	DQ118749	DQ127240	EU369083	Johnson et al. (2009), Chaverri et al. (2005)
*Neoh. pulvinatum*	P.C. 602			DQ118738	DQ118746	DQ127237		Chaverri et al. (2005)
*Neotorrubiella chinghridicola*	BCC 80733^T^	MK632039	MK632121	MK632097	MK632072	MK632176	MK632149	Thanakitpipattana et al. (2020)
*Neot. chinghridicola*	BCC 39684	MK632038	MK632122	MK632096	MK632071	MK632181	MK632148	Thanakitpipattana et al. (2020)
*Niveomyces albus*	BCC 83025^T^	ON103032		ON103157	ON125015	ON286876	ON125027	Kobmoo et al. (2023)
*Ni. albus*	BCC 73628	ON103034		ON103159	ON125017		ON125029	Kobmoo et al. (2023)
*Ni. coronatus*	NY04434800		ON493547	ON493606	ON513397	ON513399	ON513400	Araújo et al. (2022)
*Ni. formicidarum*	BCC 79346	ON103035		ON103160	ON125018	ON286878	ON125030	Kobmoo et al. (2023)
*Ni. formicidarum*	BCC 83026^T^	ON103036		ON103161	ON125019	ON286879		Kobmoo et al. (2023)
*Ni. hirsutellae*	BCC 36631^T^	ON103039		ON103164	ON125022	ON286882	ON125033	Kobmoo et al. (2023)
*Ni. hirsutellae*	BCC 36632	ON103040		ON103165	ON125023	ON286883	ON125034	Kobmoo et al. (2023)
*Ni. hirsutellae*	BCC 78482	ON103041		ON103166	ON125024	ON286884	ON125035	Kobmoo et al. (2023)
*Ni. multisynnematus*	BCC 90307	ON103037		ON103162	ON125020	ON286880	ON125031	Kobmoo et al. (2023)
*Ni. multisynnematus*	BCC 90308^T^	ON103038		ON103163	ON125021	ON286881	ON125032	Kobmoo et al. (2023)
***Ni. pseudoalbus***	**HKAS 152706^T^**	**PX900241**	**PX906584**	**PX900236**	**PX896357**	**PX896351**		**This study**
***Ni. pseudoalbus***	**HKAS 152705**	**PX900242**	**PX906585**	**PX900237**	**PX896358**	**PX896352**		**This study**
*Parahevansia koratensis*	NHJ 2662	GQ250008		GQ249982	GQ250032	ON470206	ON470208	Chuang et al. (2024)
*Para. koratensis*	NHJ 666.01	GQ250010	GQ249957	GQ249981	GQ250031			Unpublished
*Parengyodontium album*	CBS 504.83^T^	LC092880		LC092899				Tsang et al. (2016)
*Pare. album*	CBS 368.72	MH860502		MH872217	LC382183			Tsang et al. (2016), Vu et al. (2019)
*Pleurodesmospora lepidopterorum*	DY10501 ^T^	MW826576			MW834317	MW834315	MW834316	Chen et al. (2021)
*Pl. lepidopterorum*	DY10502	MW826577			MW834319		MW834318	Chen et al. (2021)
*Polystromomyces araneae*	BCC 93301^T^	MZ684101		MZ684016	MZ707825	MZ707832	MZ707845	Mongkolsamrit et al. (2022)
*Pseudogibellula formicarum*	BCC 81493	MT508781		MT512652	MT863566	MT533472		Mongkolsamrit et al. (2021)
*Pseudolecanicillium caatingaense*	URM 8447^T^			ON862926	OP290525		OP290513	Alves et al. (2022)
*Pseudol. caatingaense*	URM 8446			ON862925	OP290526		OP290514	Alves et al. (2022)
*Pseudoniveomyces arachnovorum*	BCC 95818^T^	OR098526			OR133172	OR133173	OR133174	Kobmoo et al. (2023)
*Pseudon. blattae*	BCC 53567^T^	ON103042		ON103167		ON286885	ON125036	Kobmoo et al. (2023)
*Purpureocillium lilacinum*	CBS 431.87	AY624188		EF468844	EF468791	EF468897	EF468940	Luangsa-Ard et al. (2005), Sung et al. (2007)
*Pu. lilacinum*	CBS 284.36^T^	AY624189	AY526475	FR775484	EF468792	EF468898	EF468941	Luangsa-Ard et al. (2005), Sung et al. (2007)
*Samsoniella inthanonensis*	TBRC 7916	MF140760		MF140724	MF140848	MF140789	MF140814	Mongkolsamrit et al. (2018)
*Sa. inthanonensis*	TBRC 7915^T^	MF140761		MF140725	MF140849	MF140790	MF140815	Mongkolsamrit et al. (2018)
*Simplicillium lanosoniveum*	CBS 704.86^T^	AJ292396	AF339602	AF339553	DQ522358	DQ522406	DQ522464	Sung et al. (2001), Zare et al. (2000)
*Si. lanosoniveum*	CBS 101267	AJ292395	AF339603	AF339554	DQ522357	DQ522405	DQ522463	Sung et al. (2001), Zare et al. (2000)

### Phylogenetic analyses

The assembly of forward and reverse reads was performed in BioEdit v. 7.0.5.3 (Hall et al. 2011), and initial identification was conducted through BLASTn searches in GenBank. The closely related taxa used in the phylogenetic analyses were obtained from relevant literature and downloaded from GenBank. The relevant sequence data and newly generated DNA sequences were combined, and automatic alignment and editing were completed as described by Zeng et al. (2023). The uninformative gaps and ambiguous regions of the alignments were removed using TrimAl v. 1.2. The trimmed single-locus alignments were finally concatenated with SequenceMatrix v. 1.8 (Vaidya et al. 2011). The final concatenated dataset was deposited in TreeBASE (http://purl.org/phylo/treebase/phylows/study/TB2:S30990). Maximum likelihood (ML) and Bayesian inference (BI) algorithms were used to perform phylogenetic analyses of the aligned sequences, conducted on the CIPRES Science Gateway portal (www.phylo.org) (Miller et al. 2010). The ML analysis was performed using RAxML-HPC BlackBox, with rapid bootstrap analysis followed by 1,000 bootstrap replicates; the GTR+I+G model was used for all partitions. The Bayesian analysis was used to evaluate posterior probabilities (PP) by MrBayes on XSEDE v. 3.2.7a; six simultaneous Markov chains were run for 2 million generations, with trees sampled every 200 generations, yielding 10,000 trees. The runs were terminated once convergence was achieved and the average standard deviation of split frequencies fell below 0.01. The phylogenetic trees were viewed in FigTree v. 1.4.2 (Rambaut 2016) and edited using Adobe Illustrator CS6 (Adobe Systems Inc., USA). The bootstrap support values for ML equal to or greater than 70% and PP equal to or greater than 0.90 are shown above the nodes.

## Results

### Phylogenetic analyses

**Analysis 1 *– Ophiocordycipitaceae* and *Polycephalomycetaceae***

**Figure 1.**
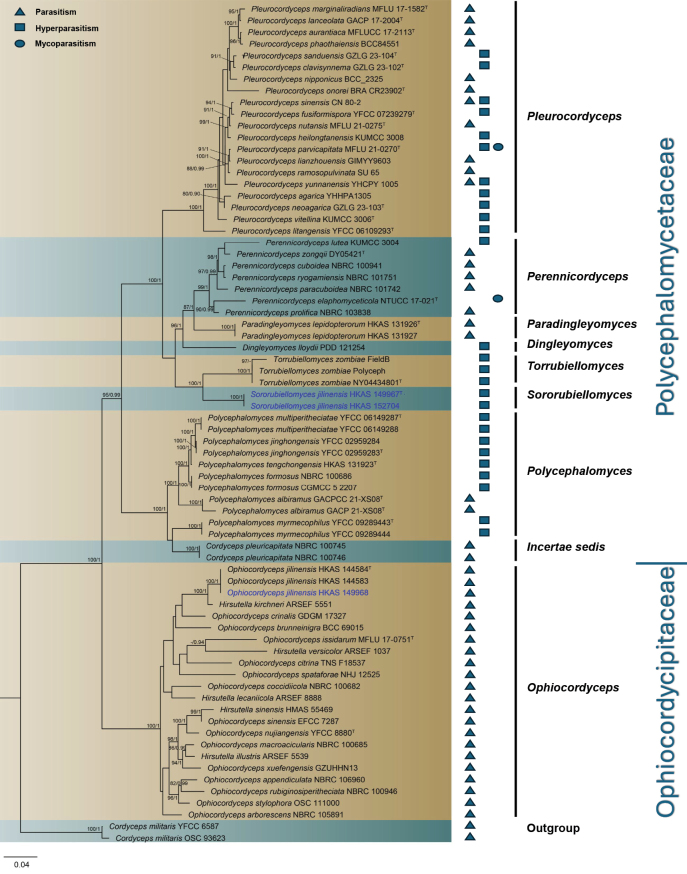
Phylogram generated from maximum likelihood analysis based on combined LSU, ITS, SSU, *tef*1-α, *rpb*1, and *rpb*2 sequence data of taxa within Ophiocordycipitaceae and Polycephalomycetaceae. ML bootstrap values equal to or greater than 70% and PP values equal to or greater than 0.90 are given above each node. Type strains are denoted by T, and sequences generated in this study are shown in blue.

A phylogenetic analysis was conducted using sequence data from six loci (LSU, ITS, SSU, *tef*1-α, *rpb*1, and *rpb*2) across 70 taxa from the families *Ophiocordycipitaceae* and *Polycephalomycetaceae*. Two strains of *Cordyceps militaris* (OSC 93623 and YFCC 6587) were designated as the outgroup. The concatenated alignment comprised 4,993 characters, including gaps, with the following distribution: LSU (836 bp), ITS (549 bp), SSU (1,016 bp), *tef*1-α (910 bp), *rpb*1 (667 bp), and *rpb*2 (1,015 bp). The best-scoring maximum likelihood tree with a log-likelihood value of –34,017.548625 is presented in Fig. 1.

This phylogenetic analysis encompassed two distinct clades: *Ophiocordyceps issidarum* and *Ophiocordyceps sinensis*, belonging to the genus *Ophiocordyceps (Ophiocordycipitaceae)*; seven genera (*Dingleyomyces*, *Paradingleyomyces*, *Perennicordyceps*, *Pleurocordyceps*, *Polycephalomyces*, *Sorobiellomyces*, and *Torrubiellomyces*) within the family *Polycephalomycetaceae*; and one *incertae sedis* taxon (*Cordyceps pleuricapitata*) within *Hypocreales*. Within this framework, the newly collected specimen *Ophiocordyceps jilinensis* (HKAS 149967) formed a clade with existing *O. jilinensis* (JL2421 and JL2420) with strong support (100% ML/1.0 PP; Fig. 1), nesting it within the broader *O. issidarum* clade. Furthermore, the new species *Sorobiellomyces jilinensis* was recovered as sister to *Torrubiellomyces zombiae* with high support (100% ML/1.0 PP; Fig. 1), confirming its placement within *Polycephalomycetaceae*.

### Analysis 2 *– Cordycipitaceae*

**Figure 2.**
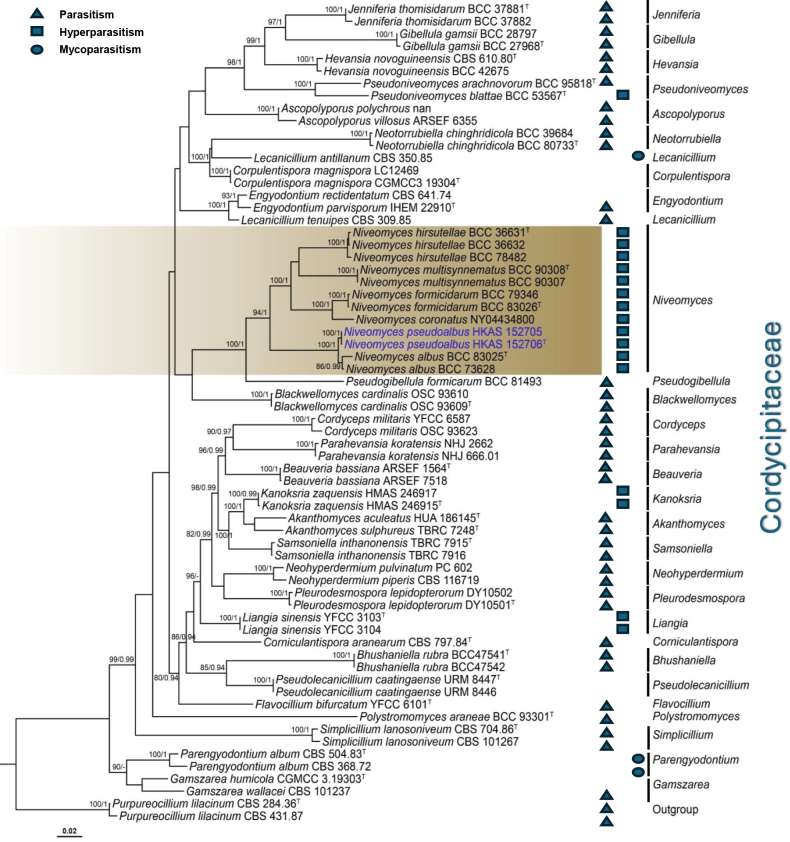
Phylogram generated from maximum likelihood analysis based on combined LSU, ITS, SSU, *tef*1-α, *rpb*1, and *rpb*2 sequence data of taxa within Cordycipitaceae. ML bootstrap values equal to or greater than 70% and PP values equal to or greater than 0.90 are given above each node. Type strains are denoted by T, and sequences generated in this study are shown in blue.

A phylogenetic analysis was conducted using sequence data from six loci (LSU, ITS, SSU, *tef*1-α, *rpb*1, and *rpb*2) across 64 taxa. Two strains of *Purpureocillium lilacinum* (CBS 284.36 and CBS 431.87) were designated as the outgroup taxa. The concatenated alignment comprised 4,701 characters, including gaps, with the following distribution: LSU (816 bp), ITS (543 bp), SSU (1,007 bp), *tef*1-α (894 bp), *rpb*1 (587 bp), and *rpb*2 (876 bp). The best-scoring maximum likelihood tree with a log-likelihood value of –40,065.681912 is presented in Fig. 2.

The phylogenetic analysis included 29 genera within the family *Cordycipitaceae* (Hyde et al. 2024), with an outgroup from *Ophiocordycipitaceae (Hypocreales)*. Within this framework, the newly discovered specimen *Niveomyces pseudoalbus* (HKAS 152706 and HKAS 152705) formed a strongly supported clade with the known *N. albus* strains (BCC 83025 and BCC 73628) (100% ML/1.0 PP; Fig. 2), confirming its placement within *Cordycipitaceae*.

### Taxonomy

#### Sorobiellomyces

Taxon classificationFungiSordariomycetesCordycipitaceae

X. Zhang, Q.F. Huang, T.C. Wen & K.D. Hyde, gen. nov.

348383C9-8456-583E-971E-83F64E522299

Index Fungorum: IF904850

##### Type.

*Sorobiellomyces jilinensis* X. Zhang, Q.F. Huang, T.C. Wen & K.D. Hyde, sp. nov.

##### Etymology.

Soro- (L.) refers to sister, indicating its phylogenetic position as the sister genus to *Torrubiellomyces*, -ubiella is derived from the name of the sister genus.

##### Description.

Hyperparasitic, parasitic on *Ophiocordyceps jilinensis*. ***Sexual morph***: Undetermined. ***Asexual morph***: The host is covered by a superficial, white to yellowish hyphal mat, with loose white hyphae present on the surface of the *Ophiocordyceps* mat. Phialides are cylindrical to subulate, frequently slightly curved, and taper gradually toward the apex, mostly branched. Conidia are unicellular, hyaline, smooth-walled, and ovoid.

##### Notes.

*Sorobiellomyces* and *Torrubiellomyces* are both monotypic genera with hosts belonging to *Ophiocordyceps* (Araújo et al. 2022). Multigene phylogenetic analysis confirms that they form distinct monophyletic lineages, supporting their status as separate genera. Morphologically, *Sorobiellomyces* is distinguished from *Torrubiellomyces* by several key characteristics of its phialides. The phialides of *Torrubiellomyces* are cylindrical to subulate, usually slightly curved, and taper gradually toward the apex. In contrast, those of *Sorobiellomyces* are consistently longer, hirsutella-like, and branched (Araújo et al. 2022). In addition, the asexual morph of *Sorobiellomyces* has been observed directly on natural specimens, whereas in species of *Torrubiellomyces*, the asexual morph has so far been reported only from cultures.

#### Sorobiellomyces jilinensis

Taxon classificationFungiSordariomycetesCordycipitaceae

X. Zhang, Q.F. Huang, T.C. Wen & K.D. Hyde, sp. nov.

99E3F16C-CBFC-52F5-A01D-2A22B3D7CC38

Index Fungorum: IF904851

Fig. 3

##### Holotype.

**China** • Jilin Province: Baishan City, Fusong County, Lushuihe Village, 42°51'N, 127°78'E, 19 August 2024, Xian Zhang, H42-1 (HKAS 149967, holotype).

**Figure 3.**
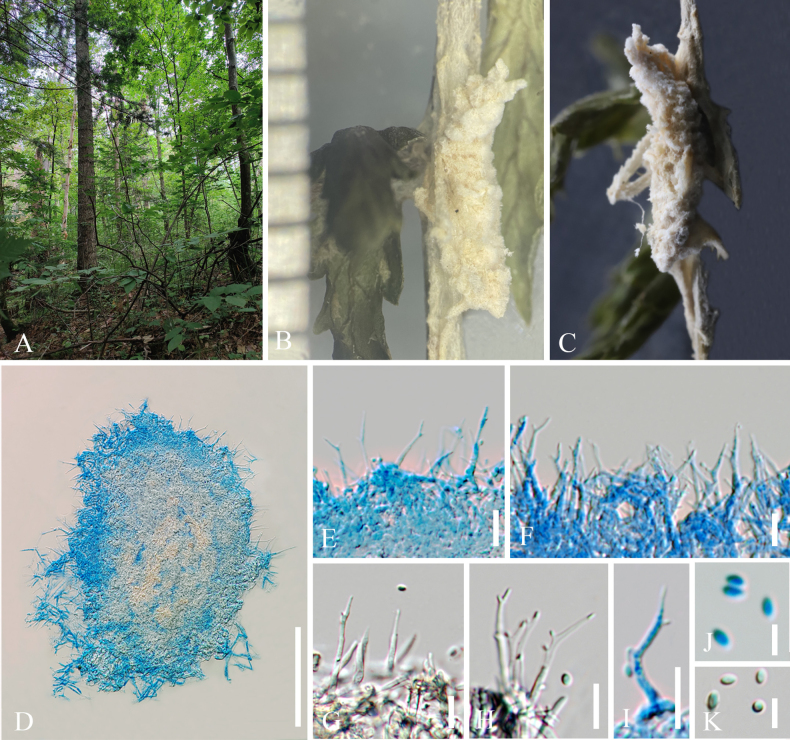
*Sorobiellomyces jilinensis* (HKAS 149967, holotype). **A**. Habitat of *Sorobiellomyces jilinensis*; **B**, **C**. Overview of *Sorobiellomyces jilinensis*; **D–F**. Cluster of phialides; **G–I**. Phialides with conidia; **J**, **K**. Conidia. Scale bars: 100 µm (**D**); 10 µm (**E–I**); 5 µm (**J, K**).

##### Etymology.

Reference to Jilin Province, the locality where the type specimen was collected.

##### Description.

Parasitic on *Ophiocordyceps jilinensis*. ***Sexual morph***: Undetermined. ***Asexual morph***: Synnemata never observed. Loose white hyphae cover the fungal hyphal mat on the insect. Phialides hirsutella-like, arising from aerial hyphae, cylindrical to subulate, base slightly swollen to inconspicuous, tapering gradually towards a narrow apex, mono- to polyphialidic, smooth, hyaline, 12.5–25.9 × 1–2.4 μm (x̄ = 18.7 × 1.8 μm, *n* = 30). Conidia 1.8–3 × 1.2–2.7 µm (x̄ = 2.4 × 1.7 µm, *n* = 30), one-celled, hyaline, smooth-walled, ovoid.

##### Habitat.

On *Ophiocordyceps jilinensis* parasitizing an insect host.

##### Additional material examined.

**China** • Jilin Province: Baishan City, Fusong County, Lushuihe Village, 42°51'N, 127°78'E, on *Ophiocordyceps jilinensis* on Orthoptera, 19 August 2024, Xian Zhang, H42-2 (HKAS 152704, paratype).

##### Notes.

The newly proposed species, *Sorobiellomyces jilinensis*, represents a previously undescribed hirsutella-like fungus that forms a strongly supported sister lineage (100% ML/1.0 PP; Fig. 1) to *Torrubiellomyces zombiae*. Ecologically, *S. jilinensis* parasitizes *Ophiocordyceps jilinensis*, whereas the type species *T. zombiae* infects the entomopathogenic fungus Ophiocordyceps camponoti-floridani. Morphologically, *S. jilinensis* lacks a known sexual morph, and its asexual morph is produced directly on the host, forming a prominent, superficial, white to yellowish hyphal mat. In contrast, *T. zombiae* produces conspicuous, blackened perithecia on the host, and its asexual morph is primarily observed in culture. A key diagnostic difference lies in the phialides of *S. jilinensis*, which are consistently longer (12.5–25.9 μm compared to 5.5–12 μm in *T. zombiae*) and explicitly noted as branched (Araújo et al. 2022). Based on this distinct phylogenetic position and unique morphological traits, *Sorobiellomyces jilinensis* is proposed as the type species of the new genus *Sorobiellomyces*.

#### Ophiocordyceps

Taxon classificationFungiSordariomycetesCordycipitaceae

Petch, Trans. Brit. Mycol. Soc. 16 (1): 74 (1931)

4D7D677A-53FE-50CF-AEC9-61CCF3257DC2

3598

##### Type.

*Ophiocordyceps blattae* (Petch) Petch, Trans. Br. mycol. Soc. 16(1): 74 (1931).

##### Description.

See Sung et al. (2007).

##### Notes.

*Ophiocordyceps* was established by Petch (1931) for species of *Cordyceps* bearing non-disarticulating ascospores, but it was subsequently treated as a subgenus by later authors (Kobayasi 1941; Mains 1958). The genus was revalidated and formally delimited through multigene phylogenetic analyses by Sung et al. (2007), who also proposed *Ophiocordycipitaceae*, with *Ophiocordyceps* as the type genus. A comprehensive revision of *Ophiocordycipitaceae* by Quandt et al. (2014) further refined the circumscription and phylogenetic placement of *Ophiocordyceps* within *Hypocreales*. Within this phylogenetic framework, the present study describes a new collection, *O. jilinensis*, supported by multilocus phylogenetic data and distinctive morphological characters.

#### Ophiocordyceps jilinensis

Taxon classificationFungiSordariomycetesCordycipitaceae

Y.P. Xiao & Y. Yang, in Cao et al., Fungal Diversity 132: 489 (2025)

74FA7FA4-C828-5DE1-A911-045DEA3583CF

Index Fungorum: IF903017

Facesoffungi Number: FoF16977

Fig. 4

##### Description.

Parasitic on adult Orthoptera. ***Sexual morph***: Undetermined. ***Asexual morph***: Hyphomycetous. Hyphae hyaline, septate, with warts. Phialides monophialidic arise laterally or terminally from the hyphae. Phialides 22.3–29.0 × 1–4.5 µm (x̄ = 26.3 × 2.4 µm, *n* = 30), with swollen base and slender neck, hyaline, cylindrical, and bear small warts. Conidia hyaline, smooth, ellipsoid to lemon shaped, arising from the apex of the neck singly, 4.5–7.1 × 4–5.1 μm (x̄ = 6.2 × 4.5 μm, *n* = 30), each enclosed within a mucous sheath.

**Figure 4.**
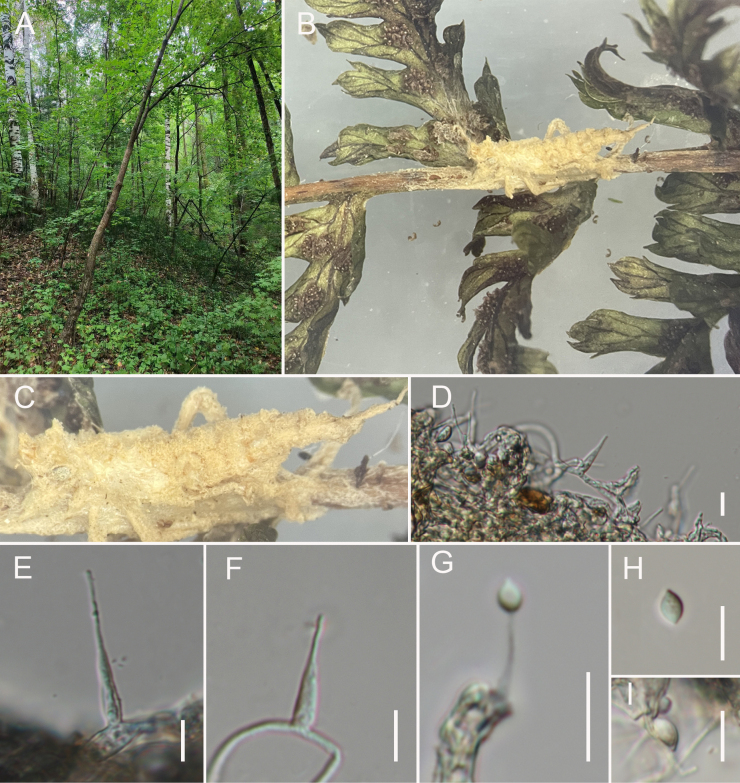
*Ophiocordyceps jilinensis* (HKAS 149968). **A**. Habitat of *O. jilinensis*; **B, C**. Overview of *O. jilinensis*; **D**. Cluster of phialides; **E, F**. Phialides; **G**. Phialides with conidia; **H, I**. Conidia. Scale bars: 10 µm (**D–G**); 5 µm (**H, I**).

##### Habitat.

Adult Orthoptera on the branches.

##### Additional material examined.

**China** • Jilin Province: Baishan City, Fusong County, Lushuihe Village, 42°33'N, 127°54'E, alt. 774 m, on adult Orthoptera, 19 August 2024, Xian Zhang, H51 (HKAS 149968).

##### Notes.

Based on a phylogenetic analysis of concatenated ITS, SSU, LSU, *tef*1-α, *rpb*1, and *rpb*2 sequences, sample HKAS 149968 grouped in a clade with the type species of *Ophiocordyceps jilinensis* (JL2421), with strong statistical support (100% ML/1.00 PP; Fig. 1). Morphologically, phialides in *Ophiocordyceps jilinensis* (JL2421) are variable, comprising both monophialidic and polyphialidic forms (Cao et al. 2025), whereas sample HKAS 149968 was observed to possess only monophialidic phialides. This difference is interpreted as intraspecific variation and does not contradict the molecular evidence.

#### Niveomyces

Taxon classificationFungiSordariomycetesCordycipitaceae

J.P.M. Araújo & de Bekker, Persoonia 49: 186 (2022)

964BC0C7-2605-50E3-9F55-EAE90EADB66C

839229

##### Type.

*Niveomyces coronatus* J.P.M. Araújo & de Bekker, Persoonia 49: 188 (2022).

##### Description.

Parasitic on *Ophiocordyceps* sp. ***Sexual morph***: Undetermined. ***Asexual morph***: *Niveomyces* species are characterized by white to pale yellow, cottony to velvety mycelium that typically covers the host. The synnemata are enveloped by a hymenium-like layer of densely arranged conidiogenous cells, which are polyblastic, cylindrical to elongated, sometimes irregular or capitate, and bear multiple crowded denticles on the apical region, forming a distinctive crown-like apex. Conidia are produced singly on the denticles and are hyaline, smooth-walled, aseptate, and varying from globose or ovoid to ellipsoidal or cylindrical depending on the species.

##### Notes.

Species of *Niveomyces* share highly similar morphological characteristics, particularly in their conidiogenous cells, which bear multiple denticles along the phialides and form a crown-like apex, producing conidia singly (Araújo et al. 2022; Kobmoo et al. 2023). Their distinctions lie mainly in conidial size and shape, which remain the primary diagnostic characters separating species within the genus (Kobmoo et al. 2023). In this study, one new species, *Niveomyces pseudoalbus*, is introduced, supported by both phylogenetic evidence and detailed morphological observations.

#### Niveomyces pseudoalbus

Taxon classificationFungiSordariomycetesCordycipitaceae

X. Zhang, T.C. Wen & K.D. Hyde sp. nov.

9C90C5EA-17FE-5378-AC0C-765A569F95F9

Index Fungorum: IF904849

Fig. 5

##### Etymology.

Reference to its close morphological resemblance to, but distinction from, its sister species *Niveomyces albus*.

**Figure 5.**
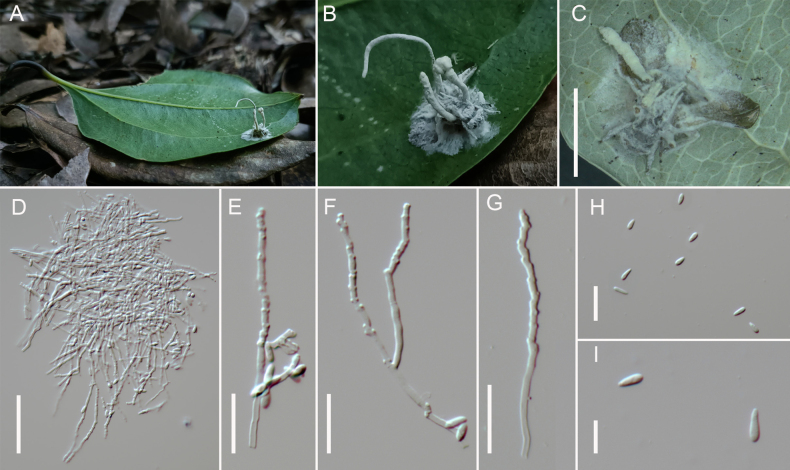
*Niveomyces pseudoalbus* (HKAS 152706, holotype). **A**. Habitat of *N. pseudoalbus*; **B**. Overview of *N. pseudoalbus*; **C**. Host; **D**. Conidiogenous cells; **E–G**. Conidiogenous cells with characteristic denticles; **H, I**. Conidia. Scale bars: 0.5 cm (**C**); 50 µm (**D**); 20 µm (**E–H**); 10 µm (**I**).

##### Holotype.

**China** • Yunnan Province: Tengchong City, Houqiao Town, 25°11'22.59"N, 98°15'53.24"E, alt. 2148 m, 18 October 2024, Xian Zhang, HQLSP1 (HKAS 125706, holotype).

##### Description.

The host is enveloped by dense, white, cottony mycelium that covers the stromata of *Ophiocordyceps dipterigena* sensu lato. ***Sexual morph***: undetermined. ***Asexual morph***: Hyphae are septate, hyaline, smooth-walled, and irregularly branched. Conidiophores are cylindrical, septate, simple, and either dichotomously or irregularly branched, with considerable variation in length. Conidiogenous cells arise directly from the hyphae, cylindrical, measuring 12.3–65.7 × 2–3.2 μm (x̄ = 33.2 × 2.5 μm, *n* = 20), and bear an irregular, geniculate rachis. Conidia are produced singly on denticles, oval to ellipsoidal with an apiculus, hyaline, smooth-walled, aseptate, and measuring 6.4–10.4 × 1.8–3.4 μm (x̄ = 7.6 × 2.7 μm, *n* = 40).

##### Habitat.

The *Ophiocordyceps dipterigena* sensu lato on the abaxial surface of leaves.

##### Additional material examined.

**China** • Yunnan Province: Tengchong City, Houqiao Town, 25°11'22.59"N, 98°15'53.24"E, alt. 2148 m, on *Ophiocordyceps dipterigena* sensu lato, 18 October 2024, Xian Zhang, HQLSP2 (HKAS 125705, paratype).

##### Notes.

Based on multilocus phylogenetic analysis, *Niveomyces pseudoalbus* (HKAS 152706 and HKAS 152705) formed a highly supported clade with *N. albus* (100% ML/1.00 PP; Fig. 2). Although these two species share similar morphology, *N. pseudoalbus* possesses more slender conidia (6.4–10.4 × 1.8–3.4 μm) than those of *N. albus* (5–7 × 1–2 μm). The conidia of *N. albus* are obtuse at both ends, whereas those of *N. pseudoalbus* are distinctly acute at one end. In addition, *N. pseudoalbus* (HKAS 152706) differs from *N. albus* (BCC 83025) by 29/834 bp (3%, 2 gaps) in LSU and 13/908 bp (1%, 0 gaps) in *tef*1-α. Overall, phylogenetic evidence supports recognizing *N. pseudoalbus* as a new species following the guidelines of Jayawardena et al. (2021).

## Discussion

Fungi represent one of the most diverse kingdoms of life, encompassing a wide range of ecological strategies and functional roles (Pineda et al. 2013; Lacey et al. 2015). Among these, some fungi parasitize other fungi, a strategy referred to as mycoparasitism or hyperparasitism (Boosalis 1964; Parratt and Laine 2016). Within *Hypocreales*, clavicipitoid fungi represent a diverse radiation of pathogens and symbionts associated with plants, insects, and other fungi (Sung et al. 2007; Kepler et al. 2012a, 2013; Xiao et al. 2023; Thiyagaraja et al. 2025). Although entomopathogenic genera such as *Metarhizium* and *Ophiocordyceps* sensu lato have been extensively studied, the ecological complexity of their associated fungal communities, particularly hyperparasitism, remains insufficiently explored.

The present work substantially expands the documented diversity of hyperparasitic fungi within this clade, delineating a total of 16 genera distributed among three families: *Polycephalomycetaceae* (six genera), *Cordycipitaceae* (eight genera), and *Clavicipitaceae* (two genera). This consolidated diversity offers a robust foundation for a comprehensive discussion of the phylogenetic breadth, ecological significance, and evolutionary trajectories of hyperparasitism within these fungi. The phylogenetic distribution of the genera reported herein confirms that hyperparasitism has evolved independently multiple times within *Hypocreales* (Wang et al. 2014; Zhong et al. 2016; Crous et al. 2017; Mongkolsamrit et al. 2021). This evolutionary strategy is not rare but is a recurrent theme, with distinct lineages emerging in different families.

Hyperparasitism is a defining characteristic of *Polycephalomycetaceae*. Species in this family, distributed across tropical to subtropical regions, frequently grow on insects or other fungi, particularly *Ophiocordyceps* species (Wang et al. 2015a, 2015b; Crous et al. 2017). Multiple hyperparasitic genera have been identified within this family, including *Dingleyomyces*, *Perennicordyceps*, *Pleurocordyceps*, *Polycephalomyces*, *Sorobiellomyces*, and *Torrubiellomyces*. This study further expands the known diversity of this ecologically versatile group by reporting the additional hyperparasitic genus *Sorobiellomyces*. Furthermore, robust phylogenetic analyses indicate that the hyperparasitic genus *Torrubiellomyces* should be transferred to *Polycephalomycetaceae*, further highlighting the ecological complexity of the family (Araújo et al. 2022; Tehan et al. 2023; Zhang et al. 2026). The finding that multiple hyperparasite species can be associated with a single *Ophiocordyceps* species pair reveals a multilayered parasitic web and hints at a wealth of undescribed diversity, solidifying the status of *Polycephalomycetaceae* as a key group for studying hyperparasitism evolution.

Within *Cordycipitaceae*, multiple genera of hyperparasitic fungi have been identified, including *Gamszarella*, *Kanoksria*, *Liangia*, *Matutinistella*, *Niveomyces*, *Pleurodesmospora*, *Pseudoniveomyces*, and *Simplicillium*. The ability of genera such as *Simplicillium* to infect both plant and insect pathogens highlights a significant diversification of this lifestyle (Wei et al. 2019). Several hyperparasitic genera have been isolated from specific fungal hosts, including *Kanoksria* from *Ophiocordyceps sinensis*, *Liangia* from *Beauveria yunnanensis*, and the monotypic *Matutinistella* from *Pseudocercospora fijiensis* (Wang et al. 2020, 2023; Khonsanit et al. 2024; Custódio and Pereira 2025). The genus *Pleurodesmospora* exhibits a broad host range, including insects, such as some araneids, mites, leafhoppers, and whiteflies; plants; and even coffee leaf rust (Samson et al. 1980; Chen et al. 2021; Yeh et al. 2021; Bu et al. 2025). Similarly, *Gamszarella* is known to parasitize insects and acts as a hyperparasite on coffee leaf rust (Crous et al. 2023; Colmán et al. 2025). Notably, *Niveomyces* and *Pseudoniveomyces* form a unique clade specialized in hyperparasitism of *Ophiocordyceps* (Araújo et al. 2020, 2022). The high host specificity observed in this genus, where different *Niveomyces* species are associated with *Ophiocordyceps* that parasitize specific insect hosts such as *Hymenoptera*, *Hemiptera*, and *Diptera* (Araújo et al. 2020, 2022; Kobmoo et al. 2023), suggests a fascinating case of coevolution or niche specialization that warrants further genomic investigation.

*Clavicipitaceae* is traditionally renowned for its plant-associated endophytes and producers of ergot alkaloids. However, the identification of hyperparasitic genera such as *Epicrea* and *Neobarya* within this family suggests that the evolutionary capacity for fungal–fungal interaction is more widespread in the clavicipitoid clade than previously recognized. For instance, *Epicrea* species have been isolated from *Hypocrella chusqueae* (Petrak 1950). Species of *Neobarya*, first described from guinea pig dung, have also been found in fungicolous and lichenicolous niches and even on *Claviceps purpurea* (Candoussau et al. 2007). This repeated niche expansion from plant symbiosis to mycoparasitism reveals an underlying evolutionary potential within the family, waiting to be unlocked by ecological opportunity.

Looking forward, this consolidated framework opens several critical avenues for future research. First, detailed field studies and co-culture experiments are needed to quantitatively assess the ecological impact of these hyperparasites on the prevalence and population dynamics of their entomopathogenic hosts. Second, genomic and metabolomic investigations of these newly reported genera will be crucial for elucidating the genetic basis of host specificity and for discovering novel antifungal compounds involved in their interfungal warfare. Finally, the potential for employing these hyperparasites as biological control agents against agricultural pests or plant diseases represents a promising applied frontier worthy of thorough exploration. In conclusion, by unveiling the extensive diversity and recurrent evolution of hyperparasitism within clavicipitoid fungi, this study not only enriches the framework for understanding fungal ecology and evolution but also opens new avenues for research in biotechnology and integrated pest management.

## Supplementary Material

XML Treatment for Sorobiellomyces

XML Treatment for Sorobiellomyces jilinensis

XML Treatment for Ophiocordyceps

XML Treatment for Ophiocordyceps jilinensis

XML Treatment for Niveomyces

XML Treatment for Niveomyces pseudoalbus

## Additional information

### Artificial Intelligence (AI) use

The authors accept full responsibility for the content of the manuscript, including the disclosure of any use of AI.

No AI tools were used in the preparation of this manuscript.

### Author ORCIDs

X. Zhang https://orcid.org/0009-0008-0919-4303

X.-C. Peng https://orcid.org/0000-0002-7271-7639

A. Mapook https://orcid.org/0000-0001-7929-2429

K.D. Hyde https://orcid.org/0000-0002-2191-0762

D.-P. Wei https://orcid.org/0000-0002-6576-2239

Z.-H. Liu https://orcid.org/0000-0001-7022-4075

N. Saichana https://orcid.org/0000-0002-4357-7980

T.-C. Wen https://orcid.org/0000-0003-1744-5869

### Data availability

All of the data that support the findings of this study are available in the main text.
